# Overexpression of Mucin 1 Suppresses the Therapeutical Efficacy of Disulfiram against Canine Mammary Tumor

**DOI:** 10.3390/ani11010037

**Published:** 2020-12-27

**Authors:** Ying Zhao, Zixiang Lin, Zhaoyan Lin, Chaoyu Zhou, Gang Liu, Jiahao Lin, Di Zhang, Degui Lin

**Affiliations:** Department of Veterinary Clinical Sciences, College of Veterinary Medicine, China Agricultural University, Beijing 100193, China; by20173050428@cau.edu.cn (Y.Z.); lzx280036@cau.edu.cn (Z.L.); bs20193050492@cau.edu.cn (Z.L.); s20193050778@cau.edu.cn (C.Z.); gangliu@cau.edu.cn (G.L.); jiahao_lin@cau.edu.cn (J.L.)

**Keywords:** canine mammary tumor, Mucin 1, disulfiram, PI3K/Akt, apoptosis

## Abstract

**Simple Summary:**

Canine mammary tumor is one of the most prevalent canine tumor types in China. Clinical studies showed that the high expression of mucin 1 (MUC1) protein is significantly associated with the malignancy and poor prognosis of canine mammary tumor. Therefore, it is worthwhile to investigate the expression of mucin 1 in developing treatments against canine mammary tumors. In the present study, it is demonstrated that disulfiram, an approved medication in treating human alcoholism, also has inhibitory effects on the growth of canine mammary tumor cells both in vitro and in vivo. With the overexpression of MUC1, the inhibitory effects of disulfiram decrease accordingly. Moreover, disulfiram is shown to inhibit phosphoinositide 3-kinase (PI3K)/serine/threonine protein kinase (Akt) signaling transduction, which is attenuated by MUC1 overexpression. Overall, these results indicate that the expression level of MUC1 is detrimental to determining the anti-tumor activity of disulfiram. Further consideration should be given when treating the canine mammary tumor with disulfiram or other PI3K/Akt inhibitors.

**Abstract:**

Mucin 1 (MUC1), a transmembrane protein, is closely associated with the malignancy and metastasis of canine mammary tumors; however, the role of overexpressed MUC1 in the development of cancer cells and response to drug treatment remains unclear. To address this question, we developed a new canine mammary tumor cell line, CIPp-MUC1, with an elevated expression level of MUC1. In vitro studies showed that CIPp-MUC1 cells are superior in proliferation and migration than wild-type control, which was associated with the upregulation of PI3K, p-Akt, mTOR, Bcl-2. In addition, overexpression of MUC1 in CIPp-MUC1 cells inhibited the suppressing activity of disulfiram on the growth and metastasis of tumor cells, as well as inhibiting the pro-apoptotic effect of disulfiram. In vivo studies, on the other side, showed more rapid tumor growth and stronger resistance to disulfiram treatment in CIPp-MUC1 xenograft mice than in wild-type control. In conclusion, our study demonstrated the importance of MUC1 in affecting the therapeutical efficiency of disulfiram against canine mammary tumors, indicating that the expression level of MUC1 should be considered for clinical use of disulfiram or other drugs targeting PI3K/Akt pathway.

## 1. Introduction

Mammary tumor is the second common type of tumor in canine species and most frequently occurred in females [1,2]. Dogs develop mammary tumors and other cancer types spontaneously with an intact immune system, which exhibits a number of clinical and molecular similarities to human breast cancer [3]. Previous studies found mucin 1 (MUC1), a transmembrane protein widely expressed in almost all glandular epithelial cells, was a protein that played an important role in the survival, proliferation, and migration of human mammary tumors. In over 90% of human breast cancer patients, overexpression of MUC1 was detected [4,5]. The fact that MUC1 overexpression was observed in various places, including primary sites, metastatic lymph nodes, migrating tumor cells, and secondary sites [6], indicated a higher risk of metastasis as well as poor prognosis of mammary tumors with the high level of MUC1 expression [7,8,9]. On the other hand, high-level expression of MUC1 led to drug resistance in mammary tumors [10]. In contrast, mouse studies showed that MUC1 deficiency effectively mitigated tumor development and metastasis [11,12], and downregulation of MUC1 expression also promoted the pro-apoptotic activity of genotoxic agents on mammary tumor cells [8]. However, there are relatively few studies related to MUC1 of canine than human. Previous studies focusing on metastatic canine mammary tumors have found that MUC1 is overexpressed in both primary and metastatic sites, as well as in metastatic lymph nodes, with a statistically significant correlation between the expression level of MUC1 and distant metastasis of tumor [13,14].

As reported, frequent *PI3CA* mutations and aberrations of PI3K-Akt pathway both happened in canine mammary tumors and human breast cancers [15]. Furthermore, the mutational hotspot of *PIK3CA* in canine mammary tumors could also be found in human breast cancers [16], which indicated the importance of PI3K signaling pathway in the development of both human breast cancer and canine mammary tumors. Studies have shown that the cytoplasmic domain on the C-terminal of MUC1 interacts with and activates PI3K downstream [4]. Activation of PI3K further promoted phosphorylation of Akt on Ser473 and activation of mTOR, which involved in regulating the growth and migration of tumor cells [4,17,18]. Therefore, the role of MUC1 in canine mammary tumors, particularly in regulating anti-drug resistance, is worthwhile to be investigated.

Disulfiram has been used clinically in the treatment of alcohol abuse and dependence for decades. Recently, studies emphasized the anti-tumor activity of disulfiram in breast cancer, pancreatic ductal adenocarcinoma, colorectal cancer, prostate cancer, etc. [19,20,21,22]. It was shown that disulfiram inhibited the proliferation and migration of tumor cells, promoted apoptosis, and reduced the resistance of tumor cells against chemotherapy and radiotherapy by interfering with different intracellular signals (PI3K/Akt, NF-kB, MAPK, p97/NPL4, ALDH, and proteosome) [23,24,25], which highlighted the role of disulfiram in inhibiting the growth and metastasis of tumors [19]. As a drug that can be administered orally, disulfiram is more pet friendly compared with traditional chemotherapies given intravenously [26]. Few studies had addressed the therapeutic role of disulfiram against canine mammary tumors, here we hypothesized that the effect of disulfiram would be compromised with the high expression of MUC1.

In the present study, we investigated the effect of overexpressed MUC1 in the development of canine mammary tumors by establishing a canine mammary tumor cell line with stable overexpression of MUC1. With this model, we further investigated the potential mechanism of disulfiram in treating canine mammary tumors. Overall, our study provided new insights into the clinical treatment of canine mammary tumors.

## 2. Materials and Methods

### 2.1. Ethics Statement

All animals were treated in strict accordance with the Guidelines for Laboratory Animal Use and Care from the Chinese Center for Disease Control and Prevention and the Rules for Medical Laboratory Animals (1998) from the Chinese Ministry of Health, under protocol CAU20151001-1, which was approved by the Animal Ethics Committee of the China Agricultural University. The approval code was AW72010202-2.

### 2.2. Cell Culture

CIPp (Graduate School of Agricultural and Life Sciences, University of Tokyo, Tokyo, Japan) [27], CMT-7364 (canine mammary tumor cell lines, Veterinary Teaching Hospital, China Agricultural University, Beijing, China) [28], and MDCK (madin-darby immortalized canine kidney cells, Cell bank of the Chinese Academy of Science, Beijing, China) were cultured in DMEM (Life Technologies Inc., Carlsbad, CA, USA) with 10% fetal bovine serum (Life Technologies Inc., Carlsbad, CA USA), penicillin (100 units/mL), and streptomycin (0.1 mg/mL) (Life Technologies Inc., Carlsbad, CA, USA). All cells were cultured at 37 °C in an atmosphere containing 5% CO_2_.

### 2.3. Cloning of Canine MUC1

Canine MUC1 gene (ENSCAFT00000026980.5) was synthesized by Beijing SYKM Gene Biotechnology Co., Ltd. (China). For plasmid construction, synthesized gene fragments and empty vector plasmid pcDNA3.1 (+) (Life Technologies Inc., Carlsbad, CA, USA) were digested by restriction enzymes EcoRI and XholI, respectively. Enzyme-digested fragments were purified and ligated together with T4 DNA ligase (Thermo Fisher Scientific, Waltham, MA, USA). The ligation product was transformed into competent *E. coli* DH5α. Clones containing recombinant plasmids (designated as pcDNA3.1-MUC1-flag) were screened and plasmids were extracted and confirmed by sequencing.

### 2.4. Construction of MUC1 Overexpressed Canine Mammary Tumor Cell Line

To establish a new cell line with stable expression of MUC1, recombinant plasmid pcDNA3.1-MUC1-flag was firstly extracted with the plasmid extraction kit (Omega, Norwalk, CT, USA). A total of 4 μg of plasmid was transfected into CIPp cells cultured in a 6-well cell culture plate (Costar, Kennebunk, ME, USA) with Lipofectamine 3000 reagent (Invitrogen, Carlsbad, CA, USA) according to the manufacturer’s instructions. Forty-eight h after transfection, cells were collected and passaged in low cell density and screened with a basic medium containing G418 for 18 generations. Stable expression of MUC1 in CIPp-MUC1 cells was verified by Western blot analysis. CIPp cells without transfection were used as negative control.

### 2.5. Western Blot Assay

Protein extracts from harvested cells or tissues were concentrated and quantified through bicinchoninic acid (BCA) analysis (Life Technologies Inc., Carlsbad, CA, USA). Equal amounts of protein were loaded on SDS-PAGE gel, transferred onto polyvinylidene fluoride (PVDF) membrane (EMD Millipore, Burlington, MA, USA), and blocked and blotted subsequently. The following primary antibodies were used for Western blot analysis: MUC1 (ab45167, Abcam, Cambridge, MA, USA, 1:1000), cleaved caspase-3 (9661T, Cell Signaling Technology, Danvers, MA, USA, 1:1000), PI3K (20584-1-AP, Proteintech, Wuhan, China, 1:700), phosphorylated Akt (Ser-473) (66444-1-AP, Proteintech, Wuhan, China, 1:2000), mTOR (20657-1-AP, Proteintech, Wuhan, China, 1:500), Bcl-2 (12789-1-AP, Proteintech, Wuhan, China, 1:1000), Bax (50599-2-lg, Proteintech, Wuhan, China, 1:7000), caspase-3 (66470-2-lg, Proteintech, Wuhan, China, 1:2000), Flag-Tag (20543-1-AP, Proteintech, Wuhan, China, 1:2000), β-Tubulin (10068-a-AP, Proteintech, Wuhan, China, 1:2000), and Gapdh (60004-1-lg, Proteintech, Wuhan, China, 1:10000). After incubating with primary antibodies and washed 4 times with tris buffered saline with tween (TBST), the membranes were incubated with secondary antibodies conjugated with horseradish peroxidase (HRP) anti-mouse/rabbit IgG (SA00001–9, SA00001–1, Proteintech, Wuhan, China, 1:5000), washed 4 times with TBST, and exposed under chemiluminescent imaging analysis system (Tanon 5200, Shanghai, China). Densitometry analysis was done by Adobe Photoshop CS6 (Adobe Systems Incorporated, Mountain View, CA, USA).

### 2.6. Colony Formation Assay

To detect the effect of overexpressed MUC1 on the growth of tumor cells, CIPp or CIPp-MUC1 cells were plated 500 cells/well in 6-well plates and further cultured at 37 °C, 5% CO_2_ for 10 days. Colony formation analysis was done by staining with 0.1% crystal violet (Solarbio, Beijing, China), and cell proliferating states were recorded by photography and counted manually. Three independent experiments were carried out in duplicate.

### 2.7. Cell Survival Rate Assays

CCK-8 assay (TransGen Biotech, Beijing, China) was used to measure cell viability. Briefly, 1 × 10^4^ cells/well were co-incubated with different dose of disulfiram (20 nM, 40 nM, 80 nM, 100 nM, 200 nM) for 24 h or 48 h. Later, the medium was changed with Fresh DMEM, then 10 μL CCK-8 was added to each well and incubated at 37 °C for 30 min with shaking, the value of OD_450nm_ of each well was obtained and recorded with a microplate reader. The experiments were carried out in triplicates and the half inhibition rate (IC_50_) values were calculated.

### 2.8. Wound Healing Assay

To measure cell migration, a scratch and wound healing assay was performed. 2 × 10^5^ cells/well were plated into 6-well cell culture plates. Twelve hours later, cells were grown to confluence and were scratched with sterile 200 μL pipette tips. After scratching, cells were washed twice with phosphate buffered solution (PBS) and incubated in DMEM (Life Technologies Inc., Carlsbad, CA, USA) with 5% fetal bovine serum (Life Technologies Inc., Carlsbad, CA, USA), penicillin (100 units/mL), and streptomycin (0.1 mg/mL) (Life Technologies Inc., Carlsbad, CA, USA). The wound in each well was recorded by a microscope camera system (CKX41, OLYMPUS, Monolith, Japan) every 12 h, and the area of open wounds was calculated by ImageJ software (version 1.8.0, National Institutes of Health, Bethesda, MD, USA) [29]. Three independent experiments were carried out in duplicate.

### 2.9. Transwell Migration and Invasion Assay

This assay was performed as previously described [30,31]. 24-well inserts (Lot NO. 09219026, Costar, NY, USA) coated with Matrigel (BD Biosciences, San Jose, CA, USA) were used for invasion assays. Briefly, cells were diluted into 2 × 10^3^ cells/well, re-suspended in 200 μL serum-free medium with or without 100 mM disulfiram, and seeded into the upper chamber, while the lower chamber was filled with complete medium (with 10% fetal bovine serum). After exposure to serum, tumor cells in the upper chamber will migrate down to the lower side. Cells attaching the bottom side of the upper chamber were stained with 0.1% crystal violet (Solarbio, Beijing, China) and counted under microscope 24 h or 48 h later. Three experiments were carried out in duplicate.

### 2.10. Deoxynucleotidyl Transferase-Mediated dUTP Nick end Labeling (TUNEL) Assay

To measure cell apoptosis, TUNEL assay was performed. Briefly, 5 × 10^4^ cells were plated on cell culture slides (Solarbio, Beijing, China) in 24-well plates until approximately 70% confluent. Cells were then treated with a basic medium or medium containing disulfiram for 24 h or 48 h. After fixation in 4% paraformaldehyde for 1 h, samples were stained following the manufacturer’s instructions (TransGen, Beijing, China). Then, the cells were washed with PBS and permeabilized with 0.1% Triton X-100 (Sigma-Aldrich, St. Louis, MO, USA) for 3 min at 4 °C. After incubation with TUNEL reagent, samples were counterstained with ProLong Gold Antifade Reagent (Solarbio, Beijing, China). Images were captured with Nikon confocal microscope. Three independent experiments were carried out in duplicate.

### 2.11. Immunohistochemistry

Xenograft tumor tissues were harvested from mice and washed with PBS. After fixation with 10% (*v*/*v*) neutral-buffered formalin (Biosharp, Beijing, China), samples were embedded in paraffin wax and sectioned into 3 μm slides. After deparaffination and antigen retrieval with Sodium Citrate-Hydrochloric acid Buffer solution, sections were incubated with primary antibodies: mTOR (20657-1-AP, Proteintech, Wuhan, China, 1:250), p-Akt (66444-1-AP, Proteintech, Wuhan, China, 1:200), cleaved caspase-3 (9661T, Cell Signaling Technology, USA, 1:400) at 4 °C overnight, followed by incubation of biotinylated secondary antibodies: anti-mouse IgG (ZB-2305, ZSGB-BIO, Beijing, China, 1:2500) or anti-rabbit IgG (ZB2301, ZSGB-BIO, Beijing, China, 1:2500) at 37 °C for 1 h. Sections were stained with diaminobenzidine (Solarbio, Beijing, China) and counterstained with hematoxylin (ZSGB-BIO, Beijing, China). Images were captured with a bright field digital microscope.

### 2.12. Immunofluorescent Microscopy

Cells (5 × 10^4^) plated on coverslips and treated with or without disulfiram (100 nM) for 48 h and were fixed in 4% paraformaldehyde solution for 20 min at room temperature and permeabilized in 0.5% Triton X-100 dissolved with PBS for 5 min. Then, the cells were washed twice with PBS, incubated with anti-MUC1 (ab45167, Abcam, Cambridge, MA, USA, 1:100) or anti-PI3K (20584-1-AP, Proteintech, Wuhan, China, 1:100) antibodies diluted in 1% bull serum albumin (BSA)/PBS for overnight at 4 °C, washed three times with PBS, and incubated with appropriate conjugated secondary antibodies diluted in PBS for 1 h at 37 °C. Following two washes with PBS, the coverslips were mounted on slides with Prolong Gold Antifade mounting reagent containing 4′,6-Diamidino-2-phenylindole dihydrochloride (DAPI) (S2110, Solarbio, Beijing, China) and stored in the dark. The slides were visualized using a Nikon Confocal microscope, and pictures were taken with NIS-Element Viewer (version 5.21.0, Nikon Instruments Inc., Shanghai, China). Three experiments were carried out in duplicate.

### 2.13. Tumor Xenograft Mouse Model

To establish a tumor xenograft mouse model, 1 × 10^5^ CIPp or CIPp-MUC1 cells were resuspended in 100 μL PBS and injected subcutaneously into a 5-week-old female null-balb/c mouse (Vital River, Beijing, China). Tumor sizes were measured with a digital caliper every other day. The volume of tumor size was calculated as follows: *V* = *L* × *W*^2^/2, of which *W* corresponds to the width and *L* to the length. Seven days later, the average volume of tumor reached ~100 mm^3^, mice were then randomly assigned into two groups and treated with or without disulfiram (50 mg/kg) daily for another 21 days. By the end of treatment, all mice were sacrificed and xenograft tumors were harvested. Four groups were set up for the tumor xenograft mouse model, including CIPp xenograft only (control), CIPp-MUC1 xenograft only, CIPp xenograft plus disulfiram treatment, and CIPp-MUC1 xenograft plus disulfiram treatment. Each group contained 5 mice.

### 2.14. Statistical Analysis

Statistical analysis was performed by using GraphPad Prism7 software (version 7, GraphPad Software Inc., San Diego, CA, USA). For two-group comparisons with Gaussian distribution, a two-tailed unpaired t-test with Welch’s correction was applied when the variances of two groups were proved equal by the *F* test. For two-group comparisons with non-Gaussian distribution, a Mann–Whitney test was applied. For multigroup comparisons with Gaussian distribution, one-way ANOVA with Tukey-Kramer’s multiple-comparison test was used after the homogeneity of variance was confirmed by Bartlett’s test. For multigroup comparisons with non-Gaussian distribution, a Kruskal–Wallis test with Dunn’s test was used. *p* values of 0.05 or less were the threshold for statistical significance. *p*-values: * *p* < 0.05; ** *p* < 0.01; *** *p* < 0.001.

## 3. Results

### 3.1. Overexpression of MUC1 Contributes to the Malignant of CIPp Cells In Vitro

To address the role of MUC1 in canine mammary tumor cells, we artificially transduced the canine *MUC1* gene into CIPp, a canine mammary tumor cell line. After G418 screening, we obtained a cell line with stable overexpression of MUC1: CIPp-MUC1. Western blot analysis showed that MUC1 expression in CIPp-MUC1 cells was substantially higher than in non-transfected control cells (Figures S1a and S4). In addition, the level of PI3K, p-Akt, and mTOR was also higher in CIPp-MUC1 cells (Figure S1b). These results suggested that overexpression of MUC1 leads to the upregulation of PI3K/Akt signaling pathway. CIPp-MUC1 and CIPp cells were used in the following experiments.

### 3.2. Overexpression of MUC1 Promotes Proliferation and Migration of CIPp Cells In Vitro

To detect the effect of MUC1 in cell proliferation, an equal number of CIPp and CIPp-MUC1 cells were seeded and cultured for 10 days. After that, the colony-forming units of CIPp-MUC1 cells were compared with CIPp cells.

**Figure 1:**
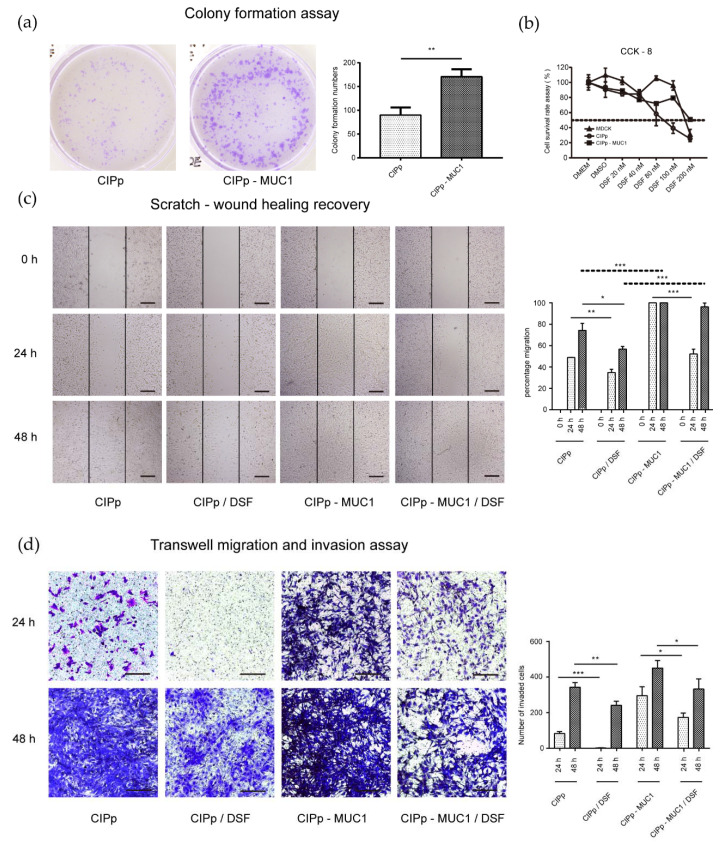
Overexpression of mucin 1 (MUC1) enhanced the proliferation and migration of canine mammary tumor cells. (**a**) 500/well CIPp or CIPp-MUC1 cells were cultured for 10 days, and colony formation was detected by crystal violet staining and counting. (**b**) CIPp, CIPp-MUC1, and MDCK cells were cultured with different concentrations of disulfiram for 48 h; cell survival rates were detected by CCK-8 staining. (**c**) CIPp and CIPp-MUC1 cells growing confluently in 6-well plates were scratched and cultured with or without 100 nM disulfiram for 24 h or 48 h. Wound healing rates were measured by dividing the newly covered area at 24 h or 48 h to the blank area at 0 h. (**d**) CIPp or CIPp-MUC1 cells were seeded on the upper chamber with serum-free medium with or without 100 nM disulfiram, and the lower chamber was filled with a complete medium. After 24 h or 48 h, the number of cells migrating through Matrigel was counted by crystal violet staining. Data were representative of three independent experiments. * *p* < 0.05, ** *p* < 0.01, *** *p* < 0.001. Bar = 100 μm.

As shown in Figure 1a, the colonies formed in CIPp-MUC1 cells were significantly more than in CIPp group, which indicated a positive effect of MUC1 in promoting cell proliferation. In addition, the rate of CIPp-MUC1 cells in wound healing was also significantly higher than CIPp cells (*p* < 0.001) (Figure 1c), which was consistent with the stronger migrating activity of CIPp-MUC1 cells observed in the transwell-migration experiment (Figure 1d). Collectively, these results indicated that overexpression of MUC1 was beneficial for the proliferation and migration of canine mammary tumor cells.

### 3.3. Overexpression of MUC1 Attenuates the Anti-Tumor Effect of Disulfiram on CIPp Cells

**Figure 2:**
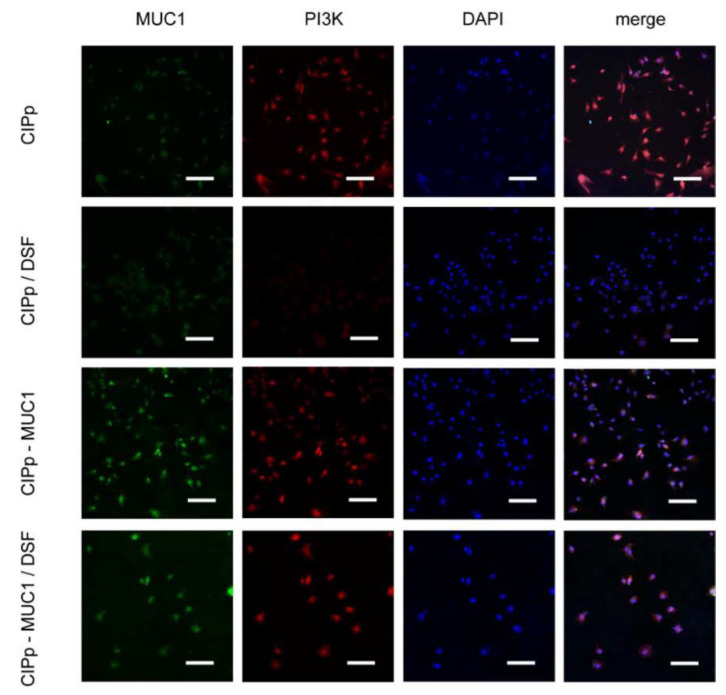
Overexpression of MUC1 attenuated the inhibitory effect of disulfiram on PI3K expression. The expression level of PI3K and MUC1 in CIPp and CIPp-MUC1 cells treated with or without 100 nM disulfiram for 48 h were detected by immunofluorescent imaging under confocal microscopy (×20). Nucleuses were stained with DAPI (Blue color, Excitation wavelength: 340 nm), PI3K proteins were stained with RFP conjugated antibody (Red color, Excitation wavelength: 550 nm), and MUC1 proteins were stained with fluorescein isothiocyanate (FITC) conjugated antibody (Green, Excitation wavelength: 488 nm). Bar = 150 μm. Data were representative of three independent experiments.

**Figure 3:**
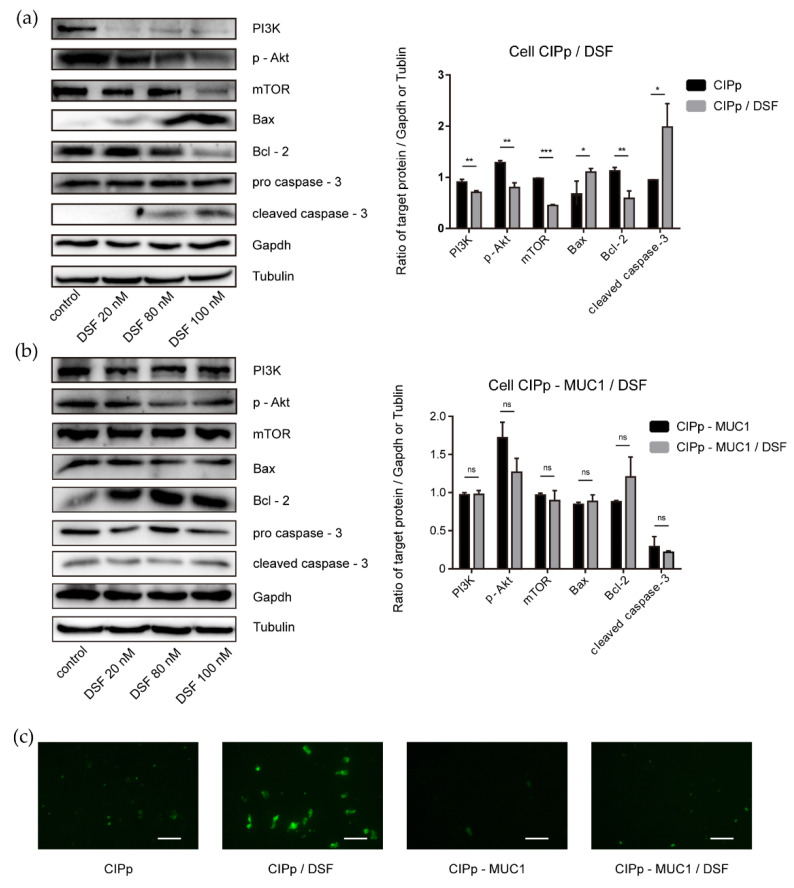
Effect of disulfiram on PI3K/Akt and apoptotic signal activity (**a**,**b**). Western blot analysis of CIPp and CIPp-MUC1 cells treated with or without different concentrations of disulfiram, the levels of PI3K, p-Akt, mTOR, Bax, Bcl-2, pro caspase-3, and cleaved caspase-3 were detected. (**a**) left panel, Western blot result of CIPp cells; right panel, densitometry analysis. (**b**) left panel, Western blot result of CIPp-MUC1 cells; right panel, densitometry analysis. (**c**) TUNEL assay of CIPp or CIPp-MUC1 cells treated with or without 100 nM disulfiram for 48 h: green signals indicated apoptotic cells (excitation wavelength: 488 nm). Data were presented as the mean ± SD of three independent experiments. * *p* < 0.05, ** *p* < 0.01, *** *p* < 0.001. Bar = 100 μm. “ns” stands for not significant.

Disulfiram was reported inhibitory against multiple signaling pathways in human breast tumor formation and progression. In our study, the potential inhibitory effect of disulfiram on CIPp cells, specifically Akt activation, was accessed. As shown in Figure 1b, the survival rate of CIPp cells was decreased to 50% with the treatment of 100 nM disulfiram for 48 h. Furthermore, we found that disulfiram treatment significantly reduced the level of PI3K, p-Akt(S473), and mTOR (Figure 2 and Figure 3a). Accordingly, the proliferation and migration of CIPp cells were largely inhibited in the presence of disulfiram (Figure 1c,d). On the other side, TUNEL results revealed that disulfiram treatment promoted cell apoptosis (Figure 3c), which is correlated with the increased level of cleaved caspase-3, Bax-2, and decreased Bcl-2 (Figure 3a) (*p* < 0.05). Meanwhile, the IC_50_ of disulfiram against CIPp cells is 97.29 ± 12.26 nM, which was lower than in CIPp-MUC1 cells (226.73 ± 16.26 nM). Overall, these results confirmed the inhibitory effect of disulfiram on PI3K/Akt/mTOR signaling pathway, which promoted apoptosis of canine mammary tumor cells.

### 3.4. Overexpression of MUC1 Promotes the Growth of Canine Mammary Tumors in Xenografted Mice

**Figure 4:**
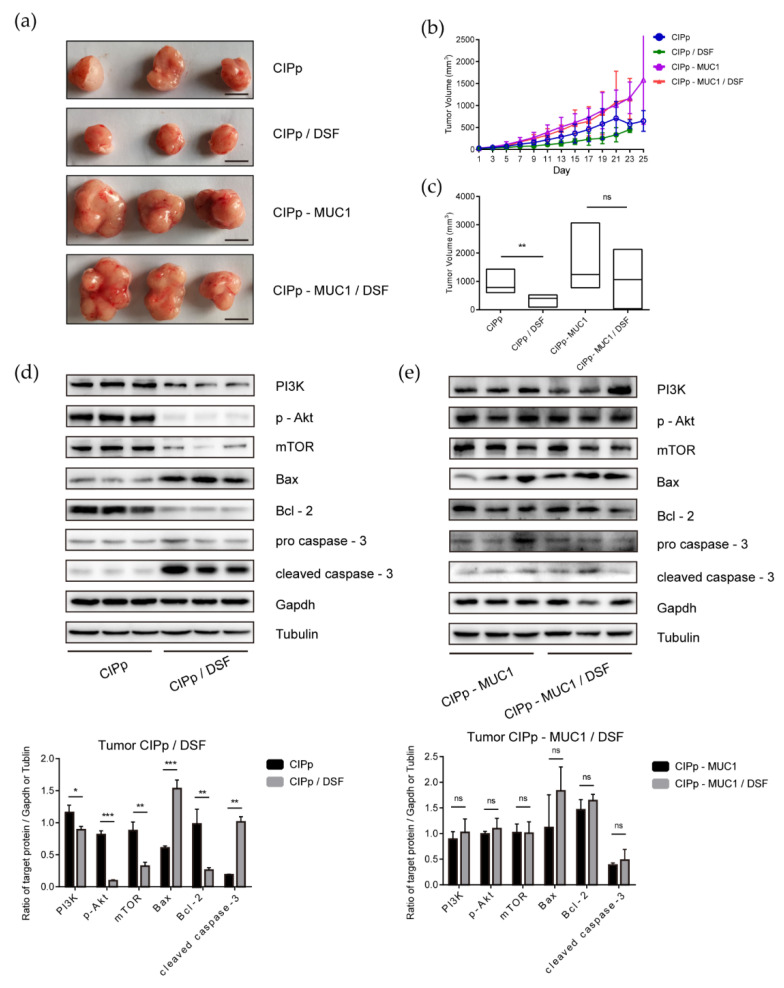
Inhibitory effect of disulfiram against CIPp or CIPp-MUC1 xenograft tumors in vivo. CIPp or CIPp-MUC1 cells were injected into mice to grow for 7 days. Later, 50 mg/kg of disulfiram was used for treatment for additional 21 days, untreated tumor-bearing mice were set as controls. Bar = 1 cm. (**a**) CIPp or CIPp-MUC1 tumors with or without disulfiram treatment. (**b**) tumor volume monitoring plot. (**c**) tumor volume of each group 21 days after disulfiram treatment. (**d**,**e**) Western blot analysis of PI3K, p-Akt, mTOR, Bax, Bcl-2, pro caspase-3, and cleaved caspase-3 of tumor tissues from different groups. (**d**) CIPp tumors with or without disulfiram treatment (100 nM). (**e**) CIPp-MUC1 tumors with or without disulfiram treatment (100 nM). Data were presented as the mean ± SD of three independent experiments. * *p* < 0.05, ** *p* < 0.01, *** *p* < 0.001. “ns” stands for not significant.

To investigate the role of overexpressed MUC1 in the development of canine mammary tumors, we established a xenograft mouse model. In this experiment, balb/c-null mice were divided into 4 groups: orthotopically injection of 1 × 10^5^ of CIPp cells (group 1); orthotopically injection of 1 × 10^5^ of CIPp-MUC1 cells (group 2); orthotopically injection with 1 × 10^5^ of CIPp cells with oral administration of disulfiram (group 3); orthotopically injection of 1 × 10^5^ of CIPp-MUC1 cells with oral administration of disulfiram (group 4). Overall, tumors were clearly seen 7 days after injection in each group, and the growth of tumors was monitored for additional 21 days. As shown, CIPp-MUC1 cells grew faster than CIPp cells in vivo (Figure 4b). Till the endpoint of monitoring (28 days after injection), the volume of CIPp-MUC1 tumors was significantly higher than those in CIPp group (*p* < 0.01) (Figure 4a,c). These results indicated that overexpression of MUC1 promotes the growth of canine mammary tumors in vivo.

In addition, we also investigated the effect of disulfiram on tumor formation in vivo. As shown in Figure 4a,c for xenografted mice with CIPp cells, disulfiram treatment significantly reduced the size of tumors as compared with untreated control. In contrast, there was little difference in CIPp-MUC1 tumor volume with or without disulfiram treatment. Western blot analysis of tumor tissues showed that the levels of PI3K, p-Akt, and mTOR in CIPp tumors were reduced with disulfiram treatment, which was associated with the up-regulation of cleaved caspase-3 (*p* < 0.05) (Figure 4d). Meanwhile, disulfiram treatment had little effect on CIPp-MUC1 tumors (Figure 4e), and the results were further confirmed by immunohistochemical analysis (Figure S2). Overall, these results demonstrated that the in vivo anti-tumor activity of disulfiram is inhibited by overexpression of MUC1.

## 4. Discussion

As a transmembrane protein with tumorigenic activities, MUC1 plays an important role in the survival, proliferation, and migration of tumor cells [6,32]. Abnormal up-regulation of MUC1 was observed in various kinds of human [8,33] and canine tumors (Figure S3) and associated with poor prognosis, drug resistance, and metastasis [7,11,34,35]. Interestingly, the overexpression of MUC1 was also commonly observed in canine malignant mammary tumors and served as a strong indicator for distant metastasis [13,14]. With little information regarding MUC1 overexpression and the progression of canine mammary tumors, we tried to investigate the potential effect of overexpressed MUC1 in both in vitro and in vivo environments. By establishing a new canine mammary tumor cell line with a stable, high level of MUC1 expression (Figure S4), we successfully demonstrated that MUC1 overexpression significantly promoted the proliferation and migration of tumor cells.

PI3K/Akt signaling pathway is critical for the growth, proliferation, survival, metastasis, and drug resistance of tumors [36,37]. Activation of PI3K/Akt promotes the formation and progression of breast cancers [17]. Inhibition of PI3K/Akt, on the contrary, can trigger the apoptosis of tumor cells [38,39]. Previous studies have shown that overexpression of MUC1 leads to the upregulation of signaling molecules involved in PI3K/Akt/mTOR pathway. Genetical depletion or drug inhibition of MUC1 would otherwise inhibit signal transduction of PI3K/Akt/mTOR [40,41]. Our study showed that MUC1 overexpression promoted the activation of PI3K/Akt, which was consistent with previous results. In addition, our data showed that with the activation of PI3K/Akt, proliferation and migration of tumor cells were also largely enhanced following MUC1 overexpression (Figure 1 and Figure 3).

It has been pointed out by previous papers that disulfiram has potential therapeutic activity against various kinds of tumors [20,23], which increases the likelihood of introducing disulfiram as auxiliary medication in preclinical and clinical studies [19,42]. Regarding the mechanism of action, it has been linked to the effects on multiple pathways including cell proliferation, metabolism, and metastasis [19], among which, Akt is considered as one of the most critical players in mediating MUC1’s effects. Our study showed that disulfiram could effectively reduce the survival rate, and significantly attenuate the healing activity and migrating potential of CIPp cells in as low as 100 nM. Biochemical and TUNEL analysis showed a pro-apoptotic activity of disulfiram on CIPp cells. Above all, our study indicated that disulfiram actively inhibits PI3K/Akt/mTOR signal, which contributed to the inhibition of the growth and migration, as well as the promotion of cell death of CIPp cells.

It has been reported that down-regulation of MUC1 attenuates Akt signal, which leads to the expression of anti-apoptotic proteins [43]. In contrast, overexpression of MUC1 would lead to increased anti-apoptotic activity of tumor cells [8,44]. Our data showed that MUC1 overexpression significantly mitigated the effects of disulfiram on tumor cells, presented by stronger capability in proliferation, migration, and less apoptosis. The effect of disulfiram on inhibiting PI3K/Akt/mTOR signaling pathway was affected as well. In combination with the previous finding that down-regulation of MUC1 would inhibit drug resistance in tumor cells, our data indicated that the level of MUC1 was critical in determining the efficiency of anti-tumor drugs targeting PI3K/Akt/mTOR signaling axis.

Our in vivo data showed that MUC1 overexpression was associated with the higher growth rate of CIPp-MUC1 tumors, which suggested that MUC1 upregulation promoted the growth of canine mammary tumors in vivo. The anti-tumor activity of disulfiram was limited in CIPp cells with overexpressed MUC1, along with little interference of disulfiram on the activation of PI3K/Akt/mTOR, indicating that the level of MUC1 affected the efficiency of disulfiram in treating canine mammary tumors.

Collectively, our data confirmed the therapeutical effect of disulfiram in canine mammary tumors, which would be beneficial for future studies and clinical practice. Importantly, our results showed that up-regulation of MUC1 would dampen the efficiency of disulfiram or other relevant drugs. Therefore, a more comprehensive analysis needs to be done before making any prescriptions. In addition, the role of MUC1 expressing level in affecting drug efficiency need to be validated with different kind of canine mammary tumors, and the molecular mechanism of interactions between MUC1 and PI3K/Akt/mTOR needs to be investigated as well.

## 5. Conclusions

1. Disulfiram inhibited proliferation and migration and promoted apoptosis of CIPp cells;
2. Overexpression of MUC1 up-regulated the signal of PI3K/Akt, which promoted the proliferation and migration of CIPp cells, and mitigated the therapeutical effects of disulfiram on CIPp cells (proliferation, migration, and apoptosis);
3. Overexpression of MUC1 promoted the growth of ClPp cells in xenografted mice and attenuated the anti-tumor activity of disulfiram in vivo.

## Data Availability

Data sharing is not applicable to this article.

## Supplementary Materials

The following are available online at https://www.mdpi.com/2076-2615/11/1/37/s1, Figure S1: Validation of CIPp-MUC1 cell line, Figure S2: Immunohistochemical analysis of the effect of disulfiram on PI3k/Akt signal in xenograft tumors, Figure S3: Western blot analysis of MUC1 in CMT-7364 or non-lactating mammary tissue, Figure S4: MUC-1 expression in different passages of CIPp-MUC1 cell line.

## References

[1] Goldschmidt M., Peña L., Rasotto R., Zappulli V. (2011). Classification and Grading of Canine Mammary Tumors. Veter. Pathol..

[2] Sleeckx N., de Rooster H., Veldhuis Kroeze E., Van Ginneken C., Van Brantegem L. (2011). Canine Mammary Tumors, an Overview. Reprod. Dom. Anim..

[3] Abdelmegeed S.M., Mohammed S. (2018). Canine mammary tumors as a model for human disease (Review). Oncol. Lett..

[4] Kufe D. (2012). MUC1-C oncoprotein as a target in breast cancer: Activation of signaling pathways and therapeutic approaches. Oncogene.

[5] Rahn J.J., Chow J.W., Horne G.J., Mah B.K., Emerman J.T., Hoffman P., Hugh J.C. (2005). MUC1 Mediates Transendothelial Migration in vitro by Ligating Endothelial Cell ICAM-1. Clin. Exp. Metastasis.

[6] Horm T.M., Schroeder J. (2013). MUC1 and metastatic cancer. Cell Adhes. Migr..

[7] Xu X., Chen W., Leng S., Padilla M.T., Saxton B., Hutt J., Tessema M., Kato K., Kim K.C., Belinsky S.A. (2017). Muc1 knockout potentiates murine lung carcinogenesis involving an epiregulin-mediated EGFR activation feedback loop. Carcinogenesis.

[8] Ren J., Agata N., Chen D., Li Y., Yu W.-H., Huang L., Raina D., Chen W., Kharbanda S., Kufe D. (2004). Human MUC1 carcinoma-associated protein confers resistance to genotoxic anticancer agents. Cancer Cell.

[9] Acres B., Limacher J.-M. (2005). MUC1 as a target antigen for cancer immunotherapy. Expert Rev. Vaccines.

[10] Merikhian P., Ghadirian R., Farahmand L., Mansouri S., Majidzadeh-A K. (2017). MUC1 induces tamoxifen resistance in estrogen receptor-positive breast cancer. Expert Rev. Anticancer. Ther..

[11] Rowse G.J., Spicer A.P., Lidner T.K., Gendler S.J. (1995). Delayed Mammary Tumor Progression in Muc-1 Null Mice. J. Biol. Chem..

[12] Besmer D.M., Curry J.M., Das Roy L., Tinder T.L., Sahraei M., Schettini J., Hwang S.-I., Lee Y.Y., Gendler S.J., Mukherjee P. (2011). Pancreatic Ductal Adenocarcinoma Mice Lacking Mucin 1 Have a Profound Defect in Tumor Growth and Metastasis. Cancer Res..

[13] Campos L.C., Silva J.O., Santos F.S., Araújo M.R., LaValle G.E., Ferreira E., Cassali G.D. (2015). Prognostic significance of tissue and serum HER2 and MUC1 in canine mammary cancer. J. Veter. Diagn. Investig..

[14] De Oliveira J.T., Pinho S.S., De Matos A.J., Hespanhol V., Reis C.A., Gärtner F. (2009). MUC1 expression in canine malignant mammary tumours and relationship to clinicopathological features. Veter. J..

[15] Lee K.-H., Hwang H.-J., Noh H.J., Shin T.-J., Cho J.-Y. (2019). Somatic Mutation of PIK3CA (H1047R) Is a Common Driver Mutation Hotspot in Canine Mammary Tumors as Well as Human Breast Cancers. Cancers.

[16] Kim T.-M., Yang I.S., Seung B.-J., Lee S., Kim D., Ha Y.-J., Seo M.-K., Kim K.-K., Kim H.S., Cheong J.-H. (2020). Cross-species oncogenic signatures of breast cancer in canine mammary tumors. Nat. Commun..

[17] Hare S.H., Harvey A. (2017). mTOR function and therapeutic targeting in breast cancer. Am. J. Cancer Res..

[18] Yan W., Ma X., Zhao X., Zhang S. (2018). Baicalein induces apoptosis and autophagy of breast cancer cells via inhibiting PI3K/AKT pathway in vivo and vitro. Drug Des. Develop. Ther..

[19] Triscott J., Pambid M.R., Dunn S.E. (2015). Concise Review: Bullseye: Targeting Cancer Stem Cells to Improve the Treatment of Gliomas by Repurposing Disulfiram. Stem Cells.

[20] Cong J., Wang Y., Zhang X., Zhang N., Liu L., Soukup K., Michelakos T., Hong T., DeLeo A., Cai L. (2017). A novel chemoradiation targeting stem and nonstem pancreatic cancer cells by repurposing disulfiram. Cancer Lett..

[21] Najlah M., Suliman A.S., Tolaymat I., Kurusamy S., Kannappan V., Elhissi A., Wang W. (2019). Development of Injectable PEGylated Liposome Encapsulating Disulfiram for Colorectal Cancer Treatment. Pharmaceutics.

[22] Majera D., Skrott Z., Bouchal J., Bartkova J., Simkova D., Gachechiladze M., Steigerova J., Kurfurstova D., Gursky J., Korinkova G. (2019). Targeting genotoxic and proteotoxic stress-response pathways in human prostate cancer by clinically available PARP inhibitors, vorinostat and disulfiram. Prostate.

[23] Wang W., McLeod H.L., Cassidy J. (2003). Disulfiram-mediated inhibition of NF-κB activity enhances cytotoxicity of 5-fluorouracil in human colorectal cancer cell lines. Int. J. Cancer.

[24] Kim J.Y., Cho Y., Oh E., Lee N., An H., Sung D., Cho T.-M., Seo J.H. (2016). Disulfiram targets cancer stem-like properties and the HER2/Akt signaling pathway in HER2-positive breast cancer. Cancer Lett..

[25] Chen D., Cui Q.C., Yang H., Dou Q.P. (2006). Disulfiram, a Clinically Used Anti-Alcoholism Drug and Copper-Binding Agent, Induces Apoptotic Cell Death in Breast Cancer Cultures and Xenografts via Inhibition of the Proteasome Activity. J. Cancer Res..

[26] Viola-Rhenals M., Patel K.R., Jaimes-Santamaria L., Wu G., Liu J., Dou Q.P. (2018). Recent Advances in Antabuse (Disulfiram): The Importance of its Metal-binding Ability to its Anticancer Activity. Curr. Med. Chem..

[27] Nakagawa T., Watanabe M., Ohashi E., Uyama R., Takauji S., Mochizuki M., Nishimura R., Ogawa H., Sugano S., Sasaki N. (2006). Cyclopedic protein expression analysis of cultured canine mammary gland adenocarcinoma cells from six tumors. Res. Vet. Sci..

[28] Zhang H., Pei S., Zhou B., Wang H., Du H., Zhang D., Lin D. (2018). Establishment and characterization of a new triple-negative canine mammary cancer cell line. Tissue Cell.

[29] Venter C., Niesler C.U. (2019). Rapid quantification of cellular proliferation and migration using ImageJ. BioTechniques.

[30] Hwang S., Shin D.M., Hong J.H. (2019). Drug Repurposing as an Antitumor Agent: Disulfiram-Mediated Carbonic Anhydrase 12 and Anion Exchanger 2 Modulation to Inhibit Cancer Cell Migration. Molecules.

[31] Diao H., Cheng N., Zhao Y., Xu H., Dong H., Thamm D.H., Zhang D., Lin D. (2019). Ivermectin inhibits canine mammary tumor growth by regulating cell cycle progression and WNT signaling. BMC Veter. Res..

[32] Stroopinsky D., Kufe D., Avigan D. (2016). MUC1 in hematological malignancies. Leuk. Lymphoma.

[33] Kufe D. (2009). Mucins in cancer: Function, prognosis and therapy. Nat. Rev. Cancer.

[34] Jing X., Liang H., Hao C., Yang X., Cui X. (2018). Overexpression of MUC1 predicts poor prognosis in patients with breast cancer. Oncol. Rep..

[35] Macha M.A., Krishn S.R., Jahan R., Banerjee K., Batra S.K., Jain M. (2015). Emerging potential of natural products for targeting mucins for therapy against inflammation and cancer. Cancer Treat. Rev..

[36] Soo H.-C., Chung F.F.-L., Lim K.-H., Yap V.A., Bradshaw T.D., Hii L.-W., Tan S.-H., See S.-J., Tan Y.-F., Leong C.-O. (2017). Cudraflavone C Induces Tumor-Specific Apoptosis in Colorectal Cancer Cells through Inhibition of the Phosphoinositide 3-Kinase (PI3K)-AKT Pathway. PLoS ONE.

[37] Zhang H., Xu H., Wang Y., Lu Z.-Y., Yu X.-F., Sui D.-Y. (2018). 20(S)-Protopanaxadiol-Induced Apoptosis in MCF-7 Breast Cancer Cell Line through the Inhibition of PI3K/AKT/mTOR Signaling Pathway. Int. J. Mol. Sci..

[38] Wells V., Mallucci L. (2009). Phosphoinositide 3-kinase targeting by the β galactoside binding protein cytokine negates akt gene expression and leads aggressive breast cancer cells to apoptotic death. Breast Cancer Res..

[39] Liang Y., Wang S., Liu J. (2019). Overexpression of Tumor Protein p53-regulated Apoptosis-inducing Protein 1 Regulates Proliferation and Apoptosis of Breast Cancer Cells through the PI3K/Akt Pathway. J. Breast Cancer.

[40] Gongsun X., Zhao Y., Jiang B., Xin Z., Shi M., Song L., Qin Q., Wang Q., Liu X. (2018). Inhibition of MUC1-C regulates metabolism by AKT pathway in esophageal squamous cell carcinoma. J. Cell. Physiol..

[41] Hiraki M., Suzuki Y., Alam M., Hinohara K., Hasegawa M., Jin C., Kharbanda S., Kufe D. (2016). MUC1-C Stabilizes MCL-1 in the Oxidative Stress Response of Triple-Negative Breast Cancer Cells to BCL-2 Inhibitors. Sci. Rep..

[42] Jiao Y., Hannafon B.N., Zhang R.R., Fung K.-M., Ding W.-Q. (2017). Docosahexaenoic acid and disulfiram act in concert to kill cancer cells: A mutual enhancement of their anticancer actions. Oncotarget.

[43] Tréhoux S., Duchêne B., Jonckheere N., Van Seuningen I. (2015). The MUC1 oncomucin regulates pancreatic cancer cell biological properties and chemoresistance. Implication of p42–44 MAPK, Akt, Bcl-2 and MMP13 pathways. Biochem. Biophys. Res. Commun..

[44] Iizuka M., Nakanishi Y., Fuchinoue F., Maeda T., Murakami E., Obana Y., Enomoto K., Tani M., Sakurai K., Amano S. (2015). Altered intracellular region of MUC 1 and disrupted correlation of polarity-related molecules in breast cancer subtypes. Cancer Sci..

