# Supplementary material for: Overexpression of Mucin 1 Suppresses the Therapeutical Efficacy of Disulfiram against Canine Mammary Tumor
Source: Animals (Basel). 2020 Dec 27;11(1):37. doi: 10.3390/ani11010037 (PMC7823863; doi:10.3390/ani11010037)
Supplement: Supplementary file 1 [file animals-11-00037-s001.pdf]

Overexpression of Mucin 1 suppresses the therapeutical efficacy of disulfiram against canine mammary tumor

Ying Zhao, Zixiang Lin, Zhaoyan Lin, Chaoyu Zhou, Gang Liu, Jiahao Lin, Di Zhang* and Degui Lin*

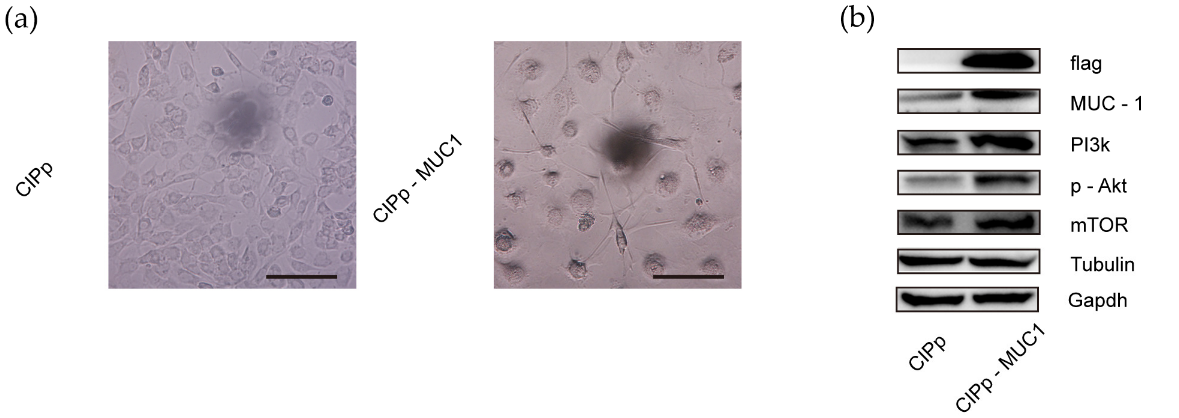

Figure S1. Validation of CIPp-MUC1 cell line. (a) Morphology or CIPp and CIPp-MUC1 cells under bright field microscope (bar= 50 μm). (b) Western blot analysis of MUC1, flag, PI3K, p-Akt, mTOR in CIPp or CIPp-MUC1 cells. Tubulin and Gapdh were set as internal control.

(a)

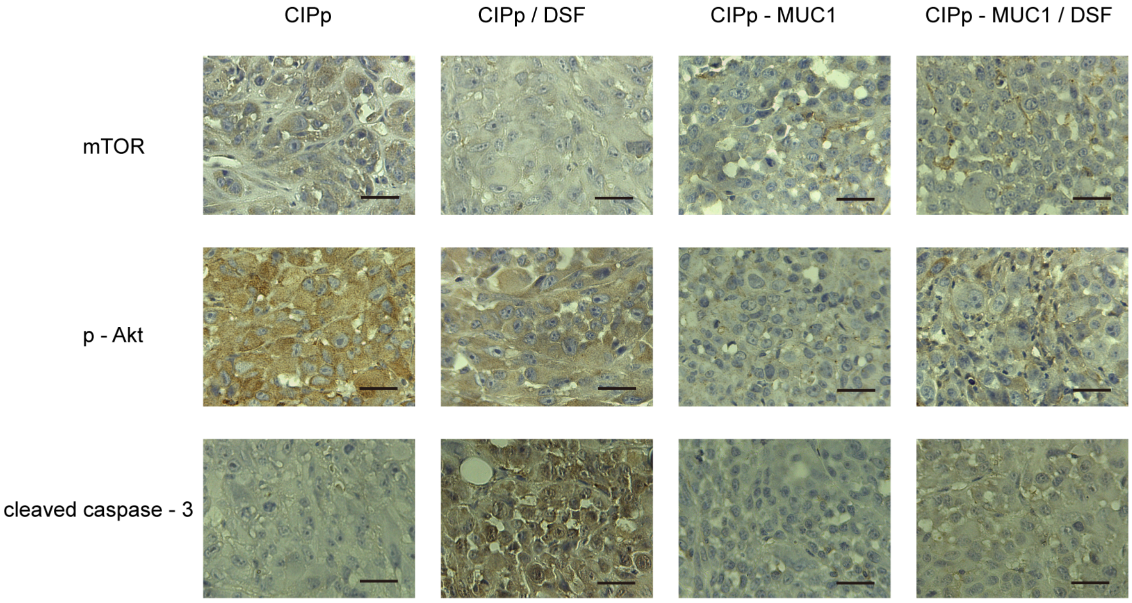

Figure S2. Immunohistochemical analysis of the effect of disulfiram on PIK3/Akt signal in xenograft tumors. (a) Immunohistochemical analysis of the level of mTOR, p-AKT and cleaved caspase-3. CIPp and CIPp-MUC1 xenograft tumors with or without 100 nM of disulfiram were compared. Brown color indicated positive signal. Bar=30 μm

(a)

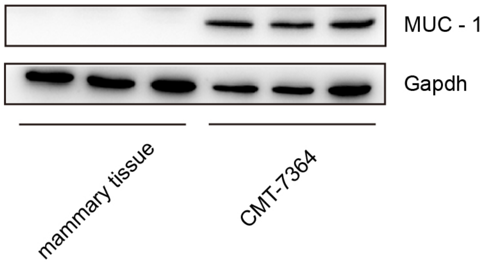

Figure S3. Western blot analysis of MUC1 in tumor formed by CMT-7364 or non-lactating mammary tissue. Gapdh was set as internal control.

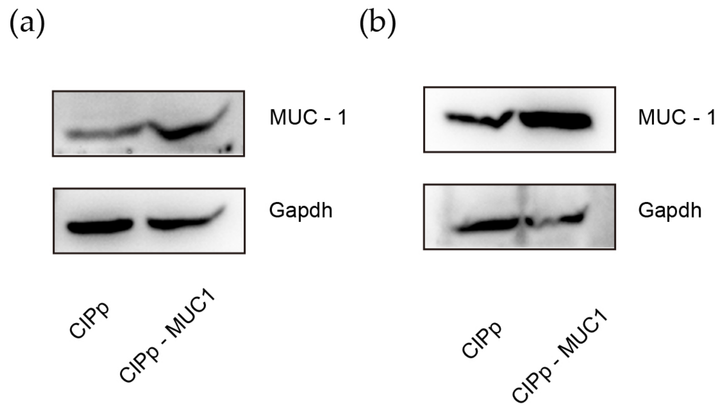

Figure S4. MUC-1 expression in different passages of CIPp-MUC1 cell line. (a) Western blot analysis of MUC1 in 10th generation of CIPp-MUC1 cells (right) and non-transfected CIPp cells (left). (b) Western blot analysis of MUC1 in 30th generation of CIPp-MUC1 cells (right) and non-transfected CIPp cells (left). Gapdh were set as internal control.

Original images of all Western blot figures

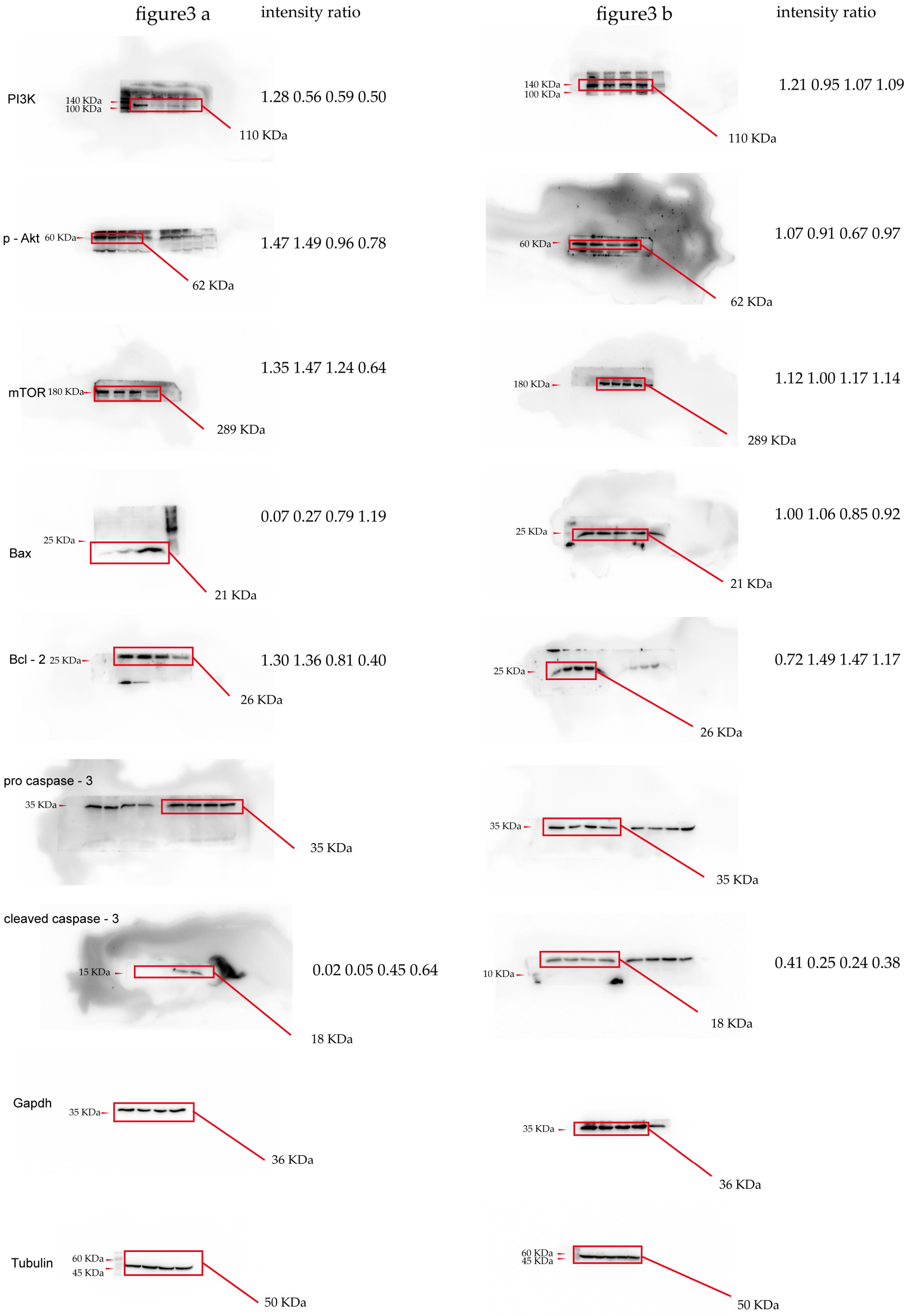

figure4 d intensity ratio figure4 e intensity ratio

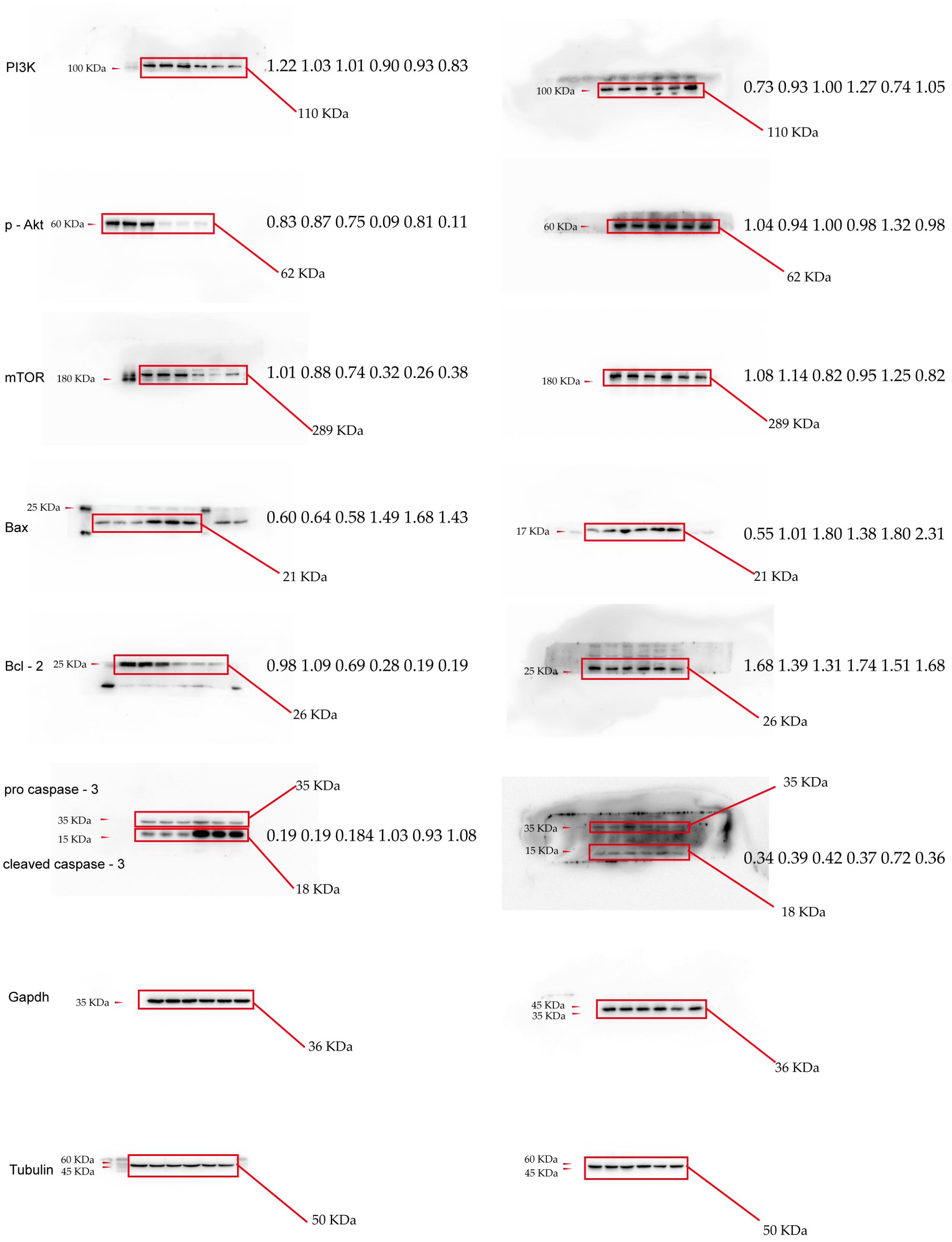

figureS 1

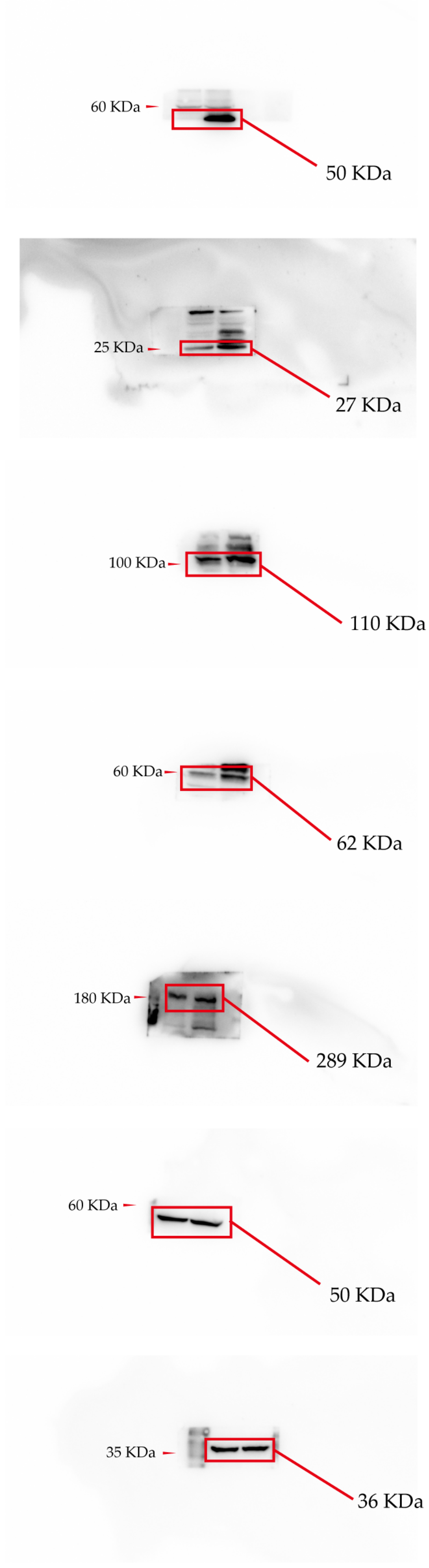

	intensity ratio
flag	0.068 1.06
MUC - 1	0.41 1.02
PI3k	1.08 1.10
p - Akt	0.40 0.87
mTOR	0.84 1.05
Tubulin	
Gapdh	

figureS 3

intensity ratio

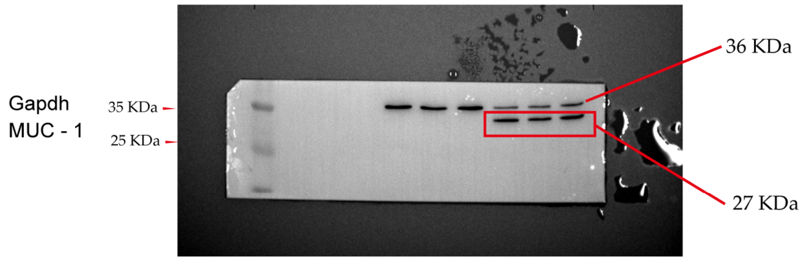

0.32 0.38 0.36 1.43 1.27

figureS 4

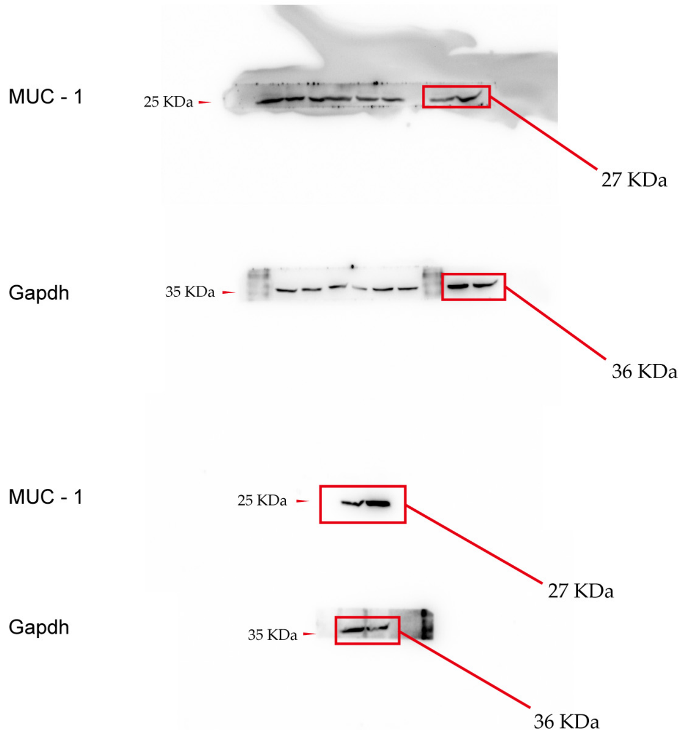

0.61 0.86

0.69 1.00
